# Comparison of oral zinc supplement and placebo effect in improving the T-cells regeneration in patients undergoing autologous hematopoietic stem cell transplantation: Clinical trial study

**DOI:** 10.1097/MD.0000000000033170

**Published:** 2024-12-20

**Authors:** Maryam Nikoonezhad, Ahmad Zavaran Hosseini, Abbas Hajifathali, Sayeh Parkhideh, Mahdi Shadnoush, Yadollah Shakiba, Hoda Zahedi

**Affiliations:** a Department of Immunology, School of Medical Sciences, Tarbiat Modares University, Tehran, Iran; b Bone Marrow Transplantation Center, Ayatollah Taleghani Hospital, Shahid Beheshti University of Medical Sciences, Tehran, Iran; c Hematopoietic Stem Cell Research Center, Shahid Beheshti University of Medical Sciences, Tehran, Iran; d Department of Clinical Nutrition, Faculty of Nutrition & Food Technology, Shahid Beheshti University of Medical Sciences, Tehran, Iran; e Regenerative Medicine Research Center, Kermanshah University of Medical Sciences, Kermanshah, Iran.

**Keywords:** hematopoietic stem cell transplantation, immune reconstitution, T-cell, thymus, zinc

## Abstract

**Background:**

Immune reconstitution is a significant factor in the success of “hematopoietic stem cell transplantation” (HSCT). Delaying the immune reconstitution increases the risk of infections and relapse after transplantation. T-cell recovery after HSCT is mainly thymus-dependent, and thymic atrophy is associated with various clinical conditions that correlate with HSCT outcomes. Thymus rejuvenation can improve immune reconstitution after transplantation.

Zinc (Zn) plays a pivotal role in thymus rejuvenation. Zn deficiency can lead to thymic atrophy, which increases susceptibility to infections. Zn supplementation restores the immune system by increasing thymus output and T-cell repertoire production.

We designed this protocol to investigate the effect of oral Zn supplementation on T-cell recovery in patients undergoing HSCT.

**Methods:**

Forty eligible candidates for autologous-HSCT will be selected. They will be randomly divided into Zn and placebo groups. Subsequently, they will receive 3 Zn or placebo tablets for the first 30 days post-HSCT (+1 to +30), followed by 1 pill or placebo for days (+31 to +90). The copy numbers of “recent thymic emigrants” T cells and “T cell Receptor Excision Circles” (TREC) will be assessed before and after the intervention in peripheral blood mononuclear cells (PBMCs). All patients will be followed up 365 days post-HSCT for relapse and infection.

**Conclusion:**

This clinical trial is the first to determine the efficiency of “Zn gluconate” as daily Supplementation in T cell recovery post-HSCT.

If successful, an available and inexpensive drug will improve immune system reconstruction after HSCT, reduce the risk of infection, particularly viral infections, and increase patient survival.

## 1. Introduction

Post-transplant immune deficiency is significantly associated with mortality and morbidity post-hematopoietic stem cell transplantation (HSCT).^[1–3]^ The innate immune cells rapidly recover after HSCT, but lymphocyte regeneration slowly occurs 1-year post-HSCT.^[4,5]^ Delay in T-cell reconstitution attributed to factors such as age-associated thymic atrophy, thymic damage due to chemotherapy, and stem cell sources.^[5–9]^ Impaired thymic recovery is associated with an increased risk of opportunistic infections and poor clinical outcomes in HSCT.^[10]^ Long-term T-cell reconstitution is mainly thymus-dependent in patients undergoing HSCT.^[7,11,12]^ Opportunistic infections and disease recurrence are significant causes of HSCT failure.^[13]^

The thymic function is essential to recent thymic emigrant (RTEs) T-cell rejuvenation, which provides a more diverse T-cell repertoire.^[11,14]^ However, T-cells are regenerated even with reduced activity in the atrophic thymus but influence poor outcomes after HSCT.^[15,16]^

Preclinical studies have investigated strategies such as growth factors and cytokines to improve immune reconstitution following HSCT, but few have progressed to clinical guidelines.^[17–19]^

Zn is a cofactor of more than 200 enzymes involved in many cellular functions, including proliferation, apoptosis, oxidative stress, immune responses, and inflammation.^[20–22]^ Zn is essential for regulating intracellular signaling pathways in innate and adaptive immunity, so Zn homeostasis is crucial for an adequate immune system function.^[23,24]^ Zn modulates the proinflammatory response by targeting “nuclear factor kappa B” (NF-k B), a master regulator of inflammatory responses.^[25]^ It also controls oxidative stress and regulates inflammatory cytokines.^[26]^ Zn deficiency elevates the inflammatory response leading to tissue damage.^[27]^ Mild Zn deficiency significantly decreases thymulin activity and T-cell production.^[28]^ Some studies confirmed the effect of Zn supplementation on thymic output and its ability to reduce the risk of infection in the elderly.^[27,29]^ Zn supplementation can reduce the incidence and severity of mucositis in patients receiving chemotherapy.^[30–33]^ A clinical study reported that high-dose Zn supplementation improved thymic output and T-cells reconstitution post HSCT in patients with multiple myeloma compared to the placebo group. This study reported Zn-related adverse effects in 5 patients (55.5%) following Zn supplementation intake that was likely multifactorial (4 patients had nausea, and 1 had diarrhea).^[34]^ However, the small sample size and high-dose Zn supplementation limited this study.^[34]^ Preclinical studies in murine models have also revealed that oral Zn supplementation improves thymus regeneration post-HSCT.^[35,36]^

Regarding the lack of evidence for Zn supplementation in improving T-cell reconstruction post-HSCT and the limitations of previous studies, this clinical study has been designed to determine the efficacy of Zn supplementation on T-cell reconstitution in patients undergoing autologous-HSCT. We will recruit 40 patients who are candidates for autologous HSCT. Eligible patients will be randomly divided into “Zn” and “placebo” groups. Zn supplementation or placebo will start on day + 1 after HSCT. The patient will receive 3 tablets daily (each contains 30 mg of elemental Zn or placebo) for 30 days, followed by 1 Zn tablet or placebo for 60 days (+31 to + 90). Copy numbers of “T-cell receptor excision circles” (TREC) and RTEs T-cells will be assessed before and after the intervention. All patients will be followed up for 365 days post-HSCT for recurrence and reactivation of cytomegalovirus (CMV) and Epstein–Barr virus (EBV) viral infection to evaluate T-cell function.

This research will also provide valuable information that can be used in allogeneic transplantation.

## 2. Materials and methods

### 2.1. Study design

**Figure 1.**
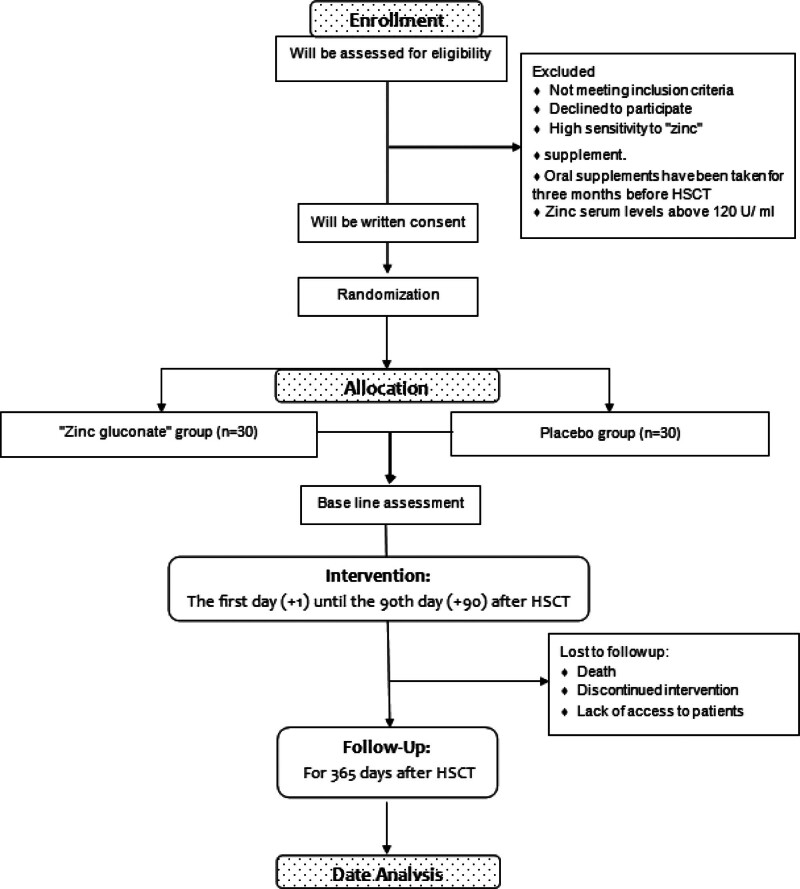
Flow diagram of the trial.

This study will be a double-blind, placebo-controlled, randomized clinical trial. In this parallel study, the candidates for autologous HSCT will randomly divide into 2 groups: Zn and placebo. The study design flow diagram is shown in Figure 1.

This study will be performed in the bone marrow transplant department of Ayatollah Taleghani Hospital, Shahid Beheshti University of Medical Sciences, and Tehran, Iran.

### 2.2. Ethics and registration

This study was approved on January 14, 2020 by the Ethical Committees of Tarbiat Modares University (IR.MODARES.REC.1398.195) and Shahid Beheshti University of Medical Sciences (IR.SBUM.REC.1399.010). It also has been registered with the Iranian Registry of Clinical Trials (IRCT20191211045701N1).

Patients who sign the consent form enter the study, a numerical code will be used for patients’ anonymity, and the interviewer will fill out a general questionnaire.

The objectives and procedures of the study will be clearly explained to patients, and they will be informed about the possible side effects of Zn supplementation. All patients will provide informed consent before enrollment, and a numerical code is dedicated to ensuring patient anonymity. Additionally, the interviewer will be filled out a general questionnaire. The participants can withdraw from the study at anytime, even after obtaining informed consent. They will not receive the fee for participating in this study and not pay extra for the drug and medical laboratory tests.

### 2.3. Patients

The subjects will be selected from the patients with “multiple myeloma” who are candidates for autologous HSCT and referred to the bone marrow transplant ward of Taleghani Hospital in Tehran. The patients will be candidates for autologous HSCT based on their medical records and the European Society for Blood and Marrow Transplantation protocols. Eligible patients would be enrolled based on the inclusion and exclusion criteria.

#### 2.3.1. Inclusion and exclusion criteria

The inclusion criteria are age between 40 and 60 years old, ability to swallow tablets, history of multiple myeloma, complete response to treatment, and a candidate for autologous HSCT without comorbidity.

Exclusion criteria will include a history of allergic reactions to oral Zn supplementations, Zn serum levels above 200 µg/dL, and taking oral Zn supplementations 3 months before intervention.

#### 2.3.2. Patient and public involvement

The research question and outcome measures were developed based on the oncologists’ experience following patients undergoing HSCT and the desire to find better immune reconstitution after HSCT. Patients and advisers were not involved in this study’s design, recruitment, or conduct. The patients or their families will be notified of the study results in writing and verbally.

### 2.4. Sample size

Ready-to-use sampling will be available based on study entry criteria. The sample size was calculated using the following formula, which is recommended for parallel clinical trials:

n = [(*z*_1_ − α/2 + *z*_1_ − β)2 · *s*_2_]/*d*_2_

A total of 40 patients who are HSCT candidates will be divided into 2 equal groups by using the block randomization method with a 95%.

### 2.5. Blinding and randomization

In this double-blind study, the researcher and participants will be blind to Zn and placebo groups. The research assistant will offer the researcher Zn and a placebo in the A and B packages to achieve this goal. A research assistant collects and provides information to the outcome assessor.

Eligible patients will be divided into 2 equal groups using block randomization. A research assistant will conduct randomization; however, the investigator, patients, staff, and outcome assessors will be blinded to the type of intervention during the study.

### 2.6. Intervention

The dosage of Zn was based on another clinical trial that used a high-dose oral Zn supplementation to improve immune reconstitution after HSCT.^[34]^ “Zn gluconate” 30 mg tablets will be purchased from Dineh Iran Industries Complex, Tehran, Iran, and placebo tablets will be prepared in similar color, shape, and weight by the Shahid Beheshti University of Medical Sciences School of Pharmacy. The placebo used in this study will be based on microcrystalline cellulose (avicel), including propylene glycol, hydroxyl propyl methyl cellulose, titanium dioxide, alcohol, and edible color. Other research assistants will pack Zn supplementations and placebos with A and B codes which will be delivered monthly to the participants. The consumption of tablets will be reminded and monitored by a text message and call phone.

The participants will be completely informed of the study objectives and asked to contact the researchers or their assistants in case of any adverse events (nausea, vomiting, diarrhea, rash, and other abnormal symptoms).^[37]^

### 2.7. Assessments

A demographic questionnaire including age, sex, education level, marital status, diagnosis, previous chemotherapy, height, and weight will be filled out for all participants before entering the study.

Two 3-day feed registration questionnaires will be filled out to ensure nondifference in food intake between the intervention and placebo groups. All people will be trained on how to complete these food records. Each patient will complete 2 “3-day feed registration” questionnaires before and after transplantation during the intervention at home. Each patient will have 6 questionnaires until the end of the study. Based on the 6 completed food records, we will determine the number of calorie intakes by counting both groups’ average macronutrients and micronutrients. The nutrient calculation will be performed by Nutritionist 4 software (First Data Bank, San Bruno) during the intervention. Patients will be monitored during intervention every week, and any occurrence of adverse events will be recorded. They will also be asked to contact the researchers or their assistants in case of any adverse events (nausea, vomiting, diarrhea, rash).^[38]^

Due to the interference of “Zn” and “copper” absorption, these 2 micronutrients were measured before and every 2 weeks during the intervention using atomic absorption spectroscopy.

#### 2.7.1. Fellowcytimetry

Blood samples will collect in the 2 mL EDTA-coated tubes 4 times, including admission time, 30th, 90th, and 180th day following HSCT. “Peripheral blood mononuclear cell” (PBMCs) will be isolated by density gradient centrifugation (Ficoll-Hypaque, Lymphodex, Inno-Train, Germany) and counted in the Neubauer chamber. After rinsing with PBS, the 10^6^ mononuclear cells will be stained by fluorochrome-conjugated anti-human monoclonal antibodies panel including CD4/CD31/CD45RA/CD45RO for 20 minutes at 4°C and will be analyzed by flow cytometry (Attune NxT Flow Cytometer).

Results of 2 populations of RTEs are expressed as percentages of CD4 CD45RA CD31 within CD4 T-cells and afterwards as the absolute numbers of cells per microliter of blood. CD45RA CD4 naive T-cells and CD45RO CD4 memory T-cells will be quantified in the same sample.^[39]^

#### 2.7.2. TREC assessment

For TREC quantification, DNA will be purified from 2 to 5 × 10^6^ PBMCs using a DNA isolation kit. Specific primers and Taqman probes will be used for TREC and TRAC as a housekeeping gene for absolute quantification of TREC numbers: TRECs forward primer (5′-CAC ATC CCT TTC AAC CAT GCT-3′) and probe (5′-FAM-ACA CCT CTG GTT TTT GTA AAG GTG CCC ACT-TAMRA-3′) and reverse primer for TRECs (5′-GCC AGC TGC AGG GTT TAG G-3′).^[40]^

Primers for the TRAC gene (forward 5-TGG CCT AAC CCT GAT CCTCTT-3, reverse 5-GGA TTT AGA GTC TCT CAG CTG GTA CAC-3) and probe (5-FAM-TCC CAC AGA TAT CCA GAA CCC TGA CCCTAMRA-3).^[41]^

Quantitative PCR will be carried out in a total volume of 25 µL containing; 500 to 1000 ng DNA, 10 to 15 µL Taqman universal PCR master mix, 900 nm each primer, and 200 nm probe for TRECs and TRAC genes, respectively, under the following conditions: 95°C for 10 minutes for polymerase activation, followed by 45 cycles of amplification (95°C for 15 seconds’ denaturation, 60°C for 1-minute annealing/elongation).

TREC and TRAC reactions will be conducted in separate wells of the same plate. The PCR reaction was performed on the 7500 Fast Real-Time PCR (Applied Bio systems). The number of TREC copies will be determined using a dilution series of plasmid TRECs-KRECs TCRAC, the pCR2.1-TOPO vector containing both signal joint T-cell receptor excision circle and TRAC fragment in T-A and Spe I acceptor sites, respectively.^[42]^ Standard dilutions of the vector from 10^6^ to 10^1^ copies were prepared and run in each set of PCR to obtain a 6-point standard curve. The TREC copy numbers will be calculated per 10^6^ PBMCs using mean values of TREC and TRAC obtained in PCR:

Mean TRECs × 10^6^/mean TRAC × 2

The absolute TREC concentration in peripheral blood (number of copies/mL) will be calculated using the following formula: (TREC copies/10^6^ PBMCs) × (PBMCs/mL)/10^6^.

#### 2.7.3. Follow-up

Relapse, CMV reactivation and EBV occurrence will be monitored within 365 days post-HSCT. Bone marrow aspirate and serum/urine protein electrophoresis in patients with multiple myeloma (MM) employ to detect relapse during follow-up.

### 2.8. Outcomes

#### 2.8.1. Primary outcomes

The primary purpose of this study is to evaluate the effects of “Zn supplementation” on improving T-cell reconstitution and thymic output post-HSCT. The numbers of RTEs T-cells and TRECs, as the thymic outputs, will be determined and compared between the 2 groups after intervention. Furthermore, we will evaluate relapse and non-relapse mortality rates within 365 days post-HSCT to assess T-cell function.

#### 2.8.2. Secondary outcomes

1. Determination and comparison of absolute lymphocyte count (ALC) post HSCT between 2 groups.
2. Determination and comparison of naïve T-cells post HSCT between 2 groups.

**Figure 2.**
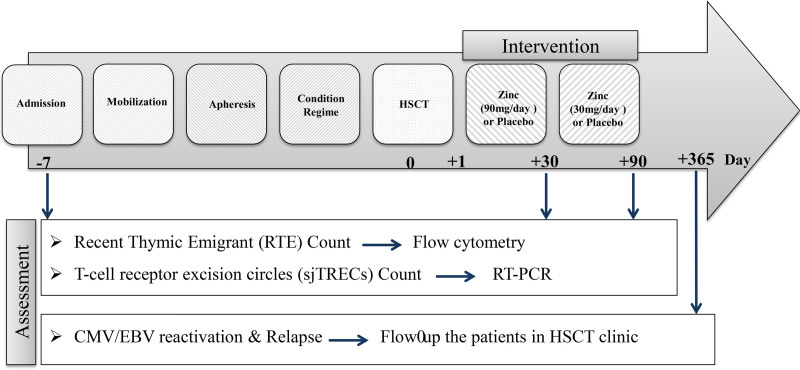
Intervention and assessment schedule of the trial. CMV = cytomegalovirus, EBV = Epstein–Bar virus, HSCT = hematopoietic stem cell transplantation, RTE = recent thymic emigrant, sjTRECs = T-cell receptor excision circles.

The intervention and assessment schedule of the trial are summarized in Figure 2.

### 2.9. Statistical analysis

SPSS version 19.0 (SPSS Inc., Chicago) will be employed for data analysis. The data entry, coding, security, and saving will be checked. Kolmogorov–Smirnov test will be used for checking normality. Wilcoxon, paired and independent *t* tests, and spearman and Pearson’s correlation coefficients will be utilized. The significant level is considered 5%. Finally, Lawrence’s method will be used to evaluate the effect of intervention groups on “non-relapse mortality” and relapse.

## 3. Discussion

After HSCT, post-transplant immune reconstitution is crucial for long-term survival.^[43,44]^ Adaptive immunity, which consists of cellular (T lymphocytes) and humoral (B lymphocytes) immunity, takes 1 to 2 years to recover after HSCT.^[5,18,44]^ Memory T-cells expand in peripheral blood and protect against previously encountered pathogens. However, the reconstitution of naïve T-cell repertoire provides immune protection against a broad range of pathogens in the long term.^[41,45]^ The thymus plays a crucial role in establishing and maintaining the appropriate microenvironment for T-cell repertoire generation under physiological and clinical conditions.^[10,46,47]^ Thymus atrophy results from aging, malnutrition, stress, and some clinical situations that decrease thymic output and naïve T-cell diversity.^[14]^ Therefore, therapeutic interventions are necessary for thymus regeneration, reducing the extent of thymic atrophy and improving the immune system.^[17,48,49]^

Studies have revealed that the improvement strategies of post-transplant T-cell reconstitution are related to improved survival in patients undergoing HSCT.^[9,50]^ However, more clinical studies are required for a specific protocol to be applied in clinical treatment.^[51,52]^

Since the role of many micronutrients, such as Zn, in immune system activities, has been proven, dietary supplementations can be investigated as safe and inexpensive drugs to improve T-cell immune reconstitution following HSCT.^[53,54]^

Zn is pivotal in many metabolic, growing pathways and immune responses.^[55]^ Immune cells require sufficient Zn to achieve a high proliferation rate, differentiation, and function.^[56]^ The immune cells rapidly react to Zn deficiency before it is measurable in the plasma.^[23]^ Zn has no specialized storage system in the body; however, a daily dose is required to maintain a steady state.^[25]^

This double-blind clinical trial is a novel study to evaluate the efficacy of “Zn gluconate” on T-cell recovery and thymic output in patients undergoing autologous HSCT. TREC and RTE T-cells produce naïve T-cells and Thymic output.^[57]^

The strengths of the trial are using a randomized, double-blind design and protocol publication, employing a safe and available strategy to improve immune reconstitution, well-absorbed and common adverse effects of Zn supplementation, and determining thymic output in autologous HSCT. Additionally, this research will provide valuable information about Immune reconstitution following HSCT that can be used in allogeneic HSCT.

## 4. Limitations

This trial has some limitations, including slow patient recruitment and long-term follow-up. In addition, this study sampling will be time-consuming due to the multiple eligibility criteria, single-center, participants’ self-reporting, dietary intakes, and lack of cooperation in some participants for completing the intervention, which leads to replacement with other patients.

## Author contributions

**Investigation:** Maryam Nikoonezhad.

**Project administration:** Ahmad Zavaran Hosseini.

**Supervision:** Abbas Hajifathali, Sayeh Parkhideh, Mahdi Shadnoush, Yadollah Shakiba, Hoda Zahedi.
